# Bacterial-Derived Outer Membrane Vesicles are Potent Adjuvants that Drive Humoral and Cellular Immune Responses

**DOI:** 10.3390/pharmaceutics13020131

**Published:** 2021-01-20

**Authors:** J. Timothy Prior, Christopher Davitt, Jonathan Kurtz, Patrick Gellings, James B. McLachlan, Lisa A. Morici

**Affiliations:** Department of Microbiology and Immunology, Tulane University School of Medicine, New Orleans, LA 70112, USA; jprior2@tulane.edu (J.T.P.); cdavitt@tulane.edu (C.D.); jkurtz@tulane.edu (J.K.); pgellings@tulane.edu (P.G.); jmclachl@tulane.edu (J.B.M.)

**Keywords:** OMVs, adjuvants, T cells, B cells, dendritic cells

## Abstract

Discovery and development of novel adjuvants that can improve existing or next generation vaccine platforms have received considerable interest in recent years. In particular, adjuvants that can elicit both humoral and cellular immune responses would be particularly advantageous because the majority of licensed vaccines are formulated with aluminum hydroxide (alum) which predominantly promotes antibodies. We previously demonstrated that bacterial-derived outer membrane vesicles (OMV) possess inherent adjuvanticity and drive antigen-specific antibody and cellular immune responses to OMV components. Here, we investigated the ability of OMVs to stimulate innate and adaptive immunity and to function as a stand-alone adjuvant. We show that OMVs are more potent than heat-inactivated and live-attenuated bacteria in driving dendritic cell activation in vitro and in vivo. Mice immunized with OMVs admixed with heterologous peptides generated peptide-specific CD4 and CD8 T cells responses. Notably, OMV adjuvant induced much greater antibody and B cell responses to co-delivered ovalbumin compared to the responses elicited by the adjuvants alum and CpG DNA. Additionally, pre-existing antibodies raised against the OMVs did not impair OMV adjuvanticity upon repeat immunization. These results indicate that vaccines adjuvanted with OMVs elicit robust cellular and humoral immune responses, supporting further development of OMV adjuvant for use in next-generation vaccines.

## 1. Introduction

Vaccination is arguably one of the greatest accomplishments in the history of public health. Vaccines using inactivated virus or live-attenuated viral strains have been tremendously successful in preventing diseases like polio, measles, and smallpox, whereas toxoid and subunit-based vaccines have been highly effective in preventing bacterial diseases like tetanus and bacterial meningitis, respectively [1]. Over the past decade, advances in nanotechnology have facilitated the development of nanoparticle-based vaccines that are approved for use in the United States and elsewhere [2,3]. For example, viral-like particles (VLP) and outer-membrane vesicles (OMV) form the basis of the FDA-licensed vaccines, Gardasil and Bexsero, for the prevention of human papilloma virus and *Neisseria meningitidis* serogroup B, respectively [4,5]. Nanoparticle-based vaccines are also in various stages of development for both re-emerging and emerging pathogens, such as *Bordetella pertussis* and SARS CoV-2 [6,7]. Immunization with nanoparticles confers several advantages including the delivery and presentation of antigens to the immune system in their native orientation as well as recognition and uptake by antigen presenting cells, including dendritic cells and B cells, leading to the development of both cellular and humoral immune responses after vaccination [8,9,10].

We were the first to show that immunization with native OMVs derived from the facultative-intracellular bacterium, *Burkholderia pseudomallei*, could provide significant protection against bacterial challenge in mice and non-human primates (NHP) [11,12,13]. Furthermore, we demonstrated that OMVs could be selectively-enriched with intracellular stage-specific proteins, leading to robust humoral and cellular immune responses that exceeded those induced by a live-attenuated strain [14]. Notably, OMVs possessed inherent adjuvanticity and conferred vaccine protection in mice and NHPs without a requirement for exogenous adjuvant. OMVs secreted by Gram-negative bacteria contain numerous pathogen-associated molecular patterns and are known to induce host pro-inflammatory responses [15]. Native, or naturally-produced, OMVs contain lipopolysaccharide (LPS), which is recognized by host Toll-like receptors (e.g., TLR4 and TLR2) and the non-canonical inflammasome via caspase-11 [16,17]. OMVs derived from bacterial species possessing a potent lipid A can induce cellular pyroptosis at high concentrations, potentially leading to sepsis [18]. Removal of LPS from OMVs by detergent extraction or genetic modification of lipid A can eliminate OMV-mediated cytotoxicity [19,20]. Like many bacterial species, *B. pseudomallei* strains differ in their lipid A composition and demonstrate considerable differences in potency. OMVs derived from *B. pseudomallei* strain 1026b or Bp82 (*purM* mutant derived from strain 1026b) possess a naturally-attenuated lipid A, which appears to confer adjuvanticity without overt cytotoxicity [21,22].

In light of our previous work, we hypothesized that Bp82-derived OMVs could function as a safe and effective vaccine adjuvant by stimulating both innate and antigen-specific adaptive immune responses. Here, we compared the relative abilities of Bp82 OMVs, heat-inactivated, and live attenuated Bp82 bacteria to activate DCs in vitro and in vivo. We also evaluated whether OMVs could elicit adaptive immunity to co-delivered antigens. We show for the first time that OMVs drive DC maturation and activation in vitro and in vivo better than or equal to whole bacteria, particularly at lower concentrations. We demonstrate that mice immunized with heterologous peptides or ovalbumin mixed with OMV adjuvant mount broad antigen-specific immune responses, including antibody, B cell, CD4 T cell, and CD8 T cells. Notably, OMV adjuvant promoted better humoral immune responses to ovalbumin compared to aluminum hydroxide (alum) and CpG DNA adjuvants. These results indicate that OMV-adjuvanted vaccines may elicit all arms of the immune response and provide a compelling basis for further development of the OMV adjuvant.

## 2. Materials and Methods

Ethics Statement: This study was carried out in accordance with recommendations from the Guide for the Care and Use of Laboratory Animals of the National Institutes of Health. Tulane University is accredited by the Association for Assessment and Accreditation of Laboratory Animal Care (AAALAC). All experimental procedures involving animals were approved and performed in compliance with the guidelines established by Tulane University School of Medicine’s Institutional Animal Care and Use Committee protocols 649 and 564.

Bacterial strains and growth conditions: *B. pseudomallei* strain Bp82 is a ∆*purM* derivative of *B. pseudomallei* strain 1026b. Bacteria were cultured from glycerol stocks immediately prior to use, and single colonies were selected from freshly streaked Luria–Bertani broth (LB) agar plates. For bacterial heat-inactivation, overnight cultures were diluted 1:100 in 50 mL of LB and grown for 6 h. The bacterial cultures were centrifuged at 9000× *g* for 10 min, and the bacterial pellets were resuspended in 5 mL dH_2_O and heat-inactivated for four hours by incubation at 60 °C. To confirm inactivation, 10% of the bacterial suspension was plated onto LB agar. A Bradford assay was used to determine protein concentration after inactivation, and heat-inactivated bacteria were stored at −20 °C until use.

OMV Purification: OMVs were purified as previously described with minor modifications [11]. Briefly, *B. pseudomallei* strain Bp82 overnight cultures were diluted 1:100 into LB and incubated at 37 °C for 16–18 h until late log phase (OD_600_ 4.5–5.0). Intact bacteria were pelleted by centrifugation (6000× *g* for 30 min at 4 °C) using an SLA-1500 fixed angle rotor. Following centrifugation, the supernatant was filtered through a 0.22 μm polyethersulfone (PES) membrane (MilliporeSigma) to remove any remaining bacteria or large bacterial fragments. Absence of bacterial contamination was verified by incubating 1 mL per liter of supernatant on LB agar for 48–72 h at 37 °C. OMVs were precipitated by incubating with 1.5 M ammonium sulfate (Fisher Scientific, Pittsburgh, PA, USA) overnight and then harvested by centrifugation (11,000× *g*, 45 min, 4 °C). Crude vesicles were resuspended in 55% sucrose (MilliporeSigma) in 30 mM Tris-HCL pH 8.0, layered at the bottom of a 35–60% density gradient, and subjected to ultracentrifugation (200,000× *g*, 3 h, 4 °C) using a 50.2Ti rotor. Fractions of equal volume were removed from the top and then evaluated by SDS-PAGE to visualize protein profiles by Coomassie blue staining [11]. Fractions containing identical protein profiles were pooled and subjected to ultracentrifugation (200,000× *g*, 19 h, 4 °C) to obtain highly purified vesicles. Purified vesicles were re-suspended in LPS-free water, visually confirmed by transmission electron microscopy, and quantitated by Bradford assay [11].

Mouse Experiments: Male and female BALB/c and C57BL/6 mice, 8 to 10 weeks old, were purchased from Charles River Laboratories (Wilmington, MA, USA) and maintained 5 per cage in polystyrene microisolator units under pathogen-free conditions. Animals were fed rodent chow and water ad libitum and allowed to acclimate 1 week prior to use.

Activation of murine dendritic cells: For in vitro experiments, bone marrow-derived dendritic cells (BMDCs) were generated from C57Bl/6 mice as described previously [23]. Cells were plated on day 10 at concentrations of 6 × 10^5^ cells/mL and stimulated 24 h later with decreasing doses of live Bp82, heat-inactivated Bp82, Bp82 OMVs, 5 ng/mL *E. coli* LPS (Sigma), or left unstimulated for 24 h. For the analysis of surface marker expression, cells were incubated with AQUA LIVE/DEAD fluorescent dye (0.5 μL) (Invitrogen) for 30 min. Cells were subsequently stained with anti-MHCcI (PE, eBioscience, San Diego, CA, USA), anti-MHCcII (eF450, eBioscience), anti-CD40 (APC, eBioscience), and anti-CD86 (BV605, BD Biosciences) and then measured by flow cytometry. For in vivo studies, C57Bl/6 mice were injected intraperitoneally with 100 μL saline control or 10 μg OMVs or bacteria that were pre-labeled with CFSE (CellTrace, Life Technologies, Carlsbad, CA, USA) according to the manufacturer’s instructions. After 6 h, cellular exudates were obtained by peritoneal lavage with 4 mL of cold PBS. Cells were stained with anti-CD11c (PE-Cy7), anti-CD11b (Alexa Fluor 700), anti-F4/80 (BV421), anti-CD40 (PE), anti-CD86 (BV605), anti-MHC class I (APC), and anti-MHC class II (PerCP Cy5.5) and analyzed by flow cytometry.

Assessment of humoral immune responses: For determination of antibody responses, mice were injected with either 10 μg of Ova, 10 μg of Ova admixed with 10 μg of CpG:alum at a 1:1 ratio, or 10 μg Ova admixed with 10 μg OMVs intramuscularly in the thigh. CpG ODN 2395 VacciGrade and Alhydrogel were purchased from InvivoGen (San Diego, CA, USA). Mice were given a second injection with each treatment 14 days after the initial injection. Seven days after the final injection, animals were humanely euthanized, and the spleen and blood were collected. Blood was collected by cardiac puncture and centrifuged in a serum separator tube (BD) at 10,000× *g* for 10 min. Sera was collected from each mouse and stored at −80 °C until analysis by enzyme linked immunosorbent assay (ELISA). For ELISA assays, U-bottom 96-well polystyrene plates (Greiner Bio-one, Kremsmünster, Austria) were coated overnight at 4 °C with Ova in coating buffer comprised of 0.1 M sodium bicarbonate +0.2 M sodium carbonate +0.1 g sodium azide in 500 mL sterile dH_2_O, at a concentration of 0.5 μg/well. Plates were then washed three times with PBS +0.5% Tween 20, hereafter referred to as PBS-T. Plates were then blocked in a 2% BSA solution in PBS-T for 1 h at room temperature. Plates were then washed three times with PBS-T. Individual mouse samples were diluted serially in a dilution buffer of 0.2% BSA solution in PBS-T. Serially diluted serum samples were added to the coated plates, and samples were incubated for 1 h at room temperature. Plates were washed three times with PBS-T. Detection by IgG ELISAs was performed using AKP-conjugated rabbit anti-mouse IgG (Thermo) as a secondary antibody, diluted 1:70,000 in 0.2% BSA solution in PBS-T, added at a volume of 100 μL/well, and then incubated for 1 h at room temperature. Plates were then washed 5 times with PBS-T. For detection, 100 μL of TMB Microwell peroxidase substrate solution (SeraCare) was added to the wells. After development of the assay, the reaction was stopped using 100 μL/well of TMB stop solution (SeraCare). The absorbance was read immediately at 450 nm to determine optical density (OD), and the data were analyzed using a sigmoidal dose–response with least-squares fit. The results were quantitated as the number of ELISA units (EU) per milliliter using the average for two sample dilutions closest to the midpoint of the standard curve.

To assess B cell responses, draining lymph nodes and spleens were combined for individual mice, and single cell suspensions were prepared by homogenizing the organs over a 100 μm nylon mesh filter in cold sorter buffer (1× phosphate buffered saline, 2% newborn calf serum, and 0.1% sodium azide). Single cell preparations were then resuspended in 50 μL of FcBlock with 1 μM PE-Cy5 decoy for 5 min at room temperature, and then 1 μM of Ova-PE B cell tetramer was added for 25 min on ice in the dark and the cells were washed. Next, the cells were stained with anti-PE magnetic beads (Miltenyi, Bergisch Gladbach, Germany) for 25 min on ice and passed over a LS column on a quadroMACS magnet. Enriched, eluted cells were stained with the following anti-mouse antibodies: GL7 (eFluor 450), CD3ε, CD11c, F4/80 (BV510), IgD (FITC), CD19 (PE-Cy7), IgM (APC), and CD38 (AF700). Cells were collected on an LSRFortessa (Beckton-Dickson). Data was analyzed using FlowJo software (TreeStar, Ashland, OR, USA). For B cell tetramer creation, we followed the protocol established by the Jenkins Lab [24] In brief, ovalbumin was biotinylated at a 1:1 ratio of biotin to protein. Next, SA-PE was added at a 6:1 ratio of protein to SA-PE to create the final tetramer. Decoy was produced in a similar manner, but an unrelated peptide (2W1S) was used as a holder, and DyLight650 was used as the fluorophore.

Assessment of Cellular Immune Responses: For measurement of CD4 and CD8 T cells, mice were injected subcutaneously with 200 μg of 2W1S or SIINFEKL peptides, respectively, with or without 1 μg of OMVs. A final group was given 1 μg OMVs plus a scrambled peptide. Fourteen days later, the mice were euthanized, and the draining lymph nodes (axial and inguinal lymph nodes) were collected to assess antigen-specific CD4 and CD8 T cells. Antigen-specific cells were enriched as previously described [24]. Briefly, cells were incubated with 10 nM of I-A^b^:2W1S (allophycocyanin conjugated) or H-2K^b^:SIINFEKL (phycoerythrin conjugated) tetramer for 1 h at room temperature in the presence of FcBlock. Samples were washed and incubated with Miltenyi (Bergisch Gladbach, Germany) anti-allophycocyanin microbeads, for 2W1S, or anti-phycoerythrin, for SIINFEKL, for 20 min on ice. Resulting samples were washed and passed over Miltenyi LS columns. The enriched, eluted cells were stained for T cell lineage negative markers (ef450; anti-CD19, anti-CD11c, anti-F4/80), anti-CD4 (BV510), anti-CD69 (BVCD69), anti-CD8 (FITC), anti-CD25 (PerCP-Cy5.5), SIINFEKL tetramer (PE), anti-CD44 (PE-Cy7), 2W1S tetramer (APC), and viability (APC-ef780) and analyzed by flow cytometry.

Statistical Analysis: Statistical differences between data sets were assessed using one-way ANOVA with Tukey’s multiple comparison test or Student *t* test on Prism (GraphPad, La Jolla, CA, USA) software. Outliers were determined using the Grubbs outlier test (ns not significant, * *p* < 0.05, ** *p* < 0.01, *** *p* < 0.001, **** *p* < 0.0001).

## 3. Results

### 3.1. Outer Membrane Vesicles (OMVs) Activate Antigen-Presenting Cells Better than Heat-Inactivated or Live-Attenuated Bacteria

**Figure 1:**
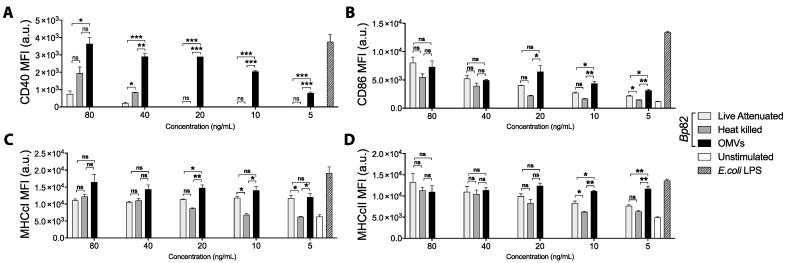
Bone marrow-derived dendritic cells (BMDCs) were stimulated for 24 h with decreasing concentrations of live or heat-killed Bp82 or outer membrane vesicles (OMVs), left unstimulated as a negative control, or 5 ng/mL *E. coli* LPS as a positive control. After incubation, cells were stained for viability and surface marker expression and analyzed by flow cytometry. BMDCs were gated as live (eF780-), CD11c+ (PE-Cy7) cells, and the expression of the maturation marker (**A**) CD40 (APC), (**B**) the co-stimulation molecule CD86 (BV605), and the antigen-presentation molecules (**C**) MHC class I (PE) and (**D**) MHC class II (eF450) were investigated. Graphs present the mean + SEM for three independent experiments. One-way ANOVA with a Tukey’s post-test were used to compare differences between groups; ns = not significant, * *p* < 0.05, ** *p* < 0.01, *** *p* < 0.001.

While both OMVs and intact bacteria are clearly recognized and taken up by antigen-presenting cells, they have never been tested head to head for their ability to drive DC activation and maturation—a critical first step in promoting cellular immunity. Here, we examined the relative abilities of OMVs, heat-inactivated, and live-attenuated bacteria to drive DC maturation and activation. For these experiments, we used heat-inactivated and live attenuated Bp82 bacteria, from which the OMVs were derived. As shown in Figure 1, OMVs exhibited a dose-dependent ability to drive DC maturation that significantly exceeded the ability of heat-killed or live strains, and the effect was sustained at OMV concentrations as low as 5 ng/mL (Figure 1A). Expression of co-stimulatory molecule CD86 and major histocompatibility marker class I (MHCcI) increased similarly in response to OMVs and bacteria at high concentrations, yet OMVs induced significantly greater expression of CD86 and MHCcI than the bacteria at lower concentrations (20 ng/mL or less) (Figure 1B,C). The same was true for MHC class II (MHCcII) at concentrations of 10 ng/mL or less (Figure 1D).

**Figure 2:**
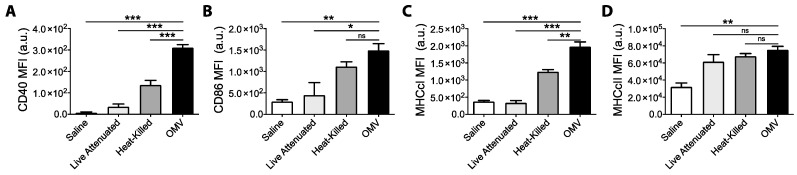
Mice (*n* = 3) were intra-peritoneally administered either with saline or 10 μg live bacteria, heat-killed bacteria, or OMVs. Peritoneal exudate cells (PerC) were recovered 6 h later by peritoneal washing and stained for viability and surface marker expression, and samples were analyzed by flow cytometry. PerC dendritic cells (DCs) were gated as live (eF780-), Dump- (CD19, B220, CD3, NK1.1) (redFlour710), CD11b+ (BV510), FSc lo, CD11c+ (PE-Cy7), and F4/80- (BV421). Median fluorescence intensity (MFI) for surface marker expression of (**A**) CD40-PE, (**B**) CD86-BV605, (**C**) MHCcI-APC, and (**D**) MHCcII-PerCP-Cy5.5 were compared between groups. Graphs present MFI data mean + SEM for 3 mice per group. One-way ANOVA with Tukey’s post-test was used to compare variances; ns not significant, * *p* < 0.05, ** *p* < 0.01, *** *p* < 0.001. Results shown are representative of two independent experiments.

To confirm our in vitro observations, we next assessed the relative abilities of OMVs and bacteria to activate DCs in vivo. Mice were administered equal doses of OMV or bacteria by intraperitoneal injection, and peritoneal cellular exudate was harvested six hours later from euthanized mice. As shown in Figure 2, peritoneal DCs harvested from mice treated with OMVs had significantly higher surface expression of CD40 and MHCcI than DCs obtained from mice treated with dead or live bacteria (Figure 2A,C). CD86 expression was also significantly higher in OMV-treated mice compared live, but not dead, bacteria treatment (Figure 2B). MHCcII was also significantly up-regulated on DCs from OMV-treated mice compared to saline-treated controls (Figure 2D). Interestingly, live bacteria induced the weakest response in vivo, which may be due to immunomodulation or suppression of host-inflammatory responses [25]. Taken together with our in vitro results, these data suggest that bacterial OMVs are more potent and more rapid activators of dendritic cells than intact bacteria.

### 3.2. OMV Adjuvant Promotes Antigen-Specific CD4 and CD8 T Cells to Co-Delivered Peptides

**Figure 3:**
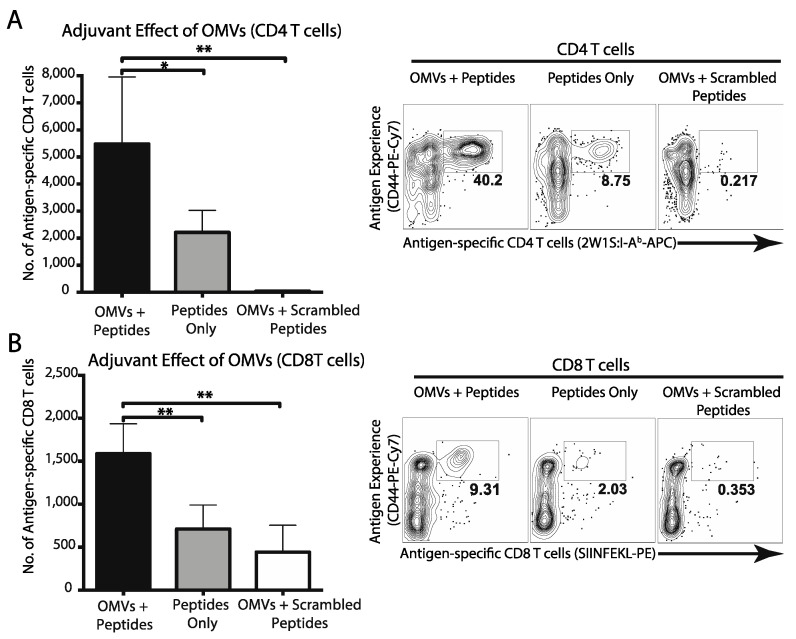
Mice (*n* = 3) were immunized subcutaneously with 200 μg of both CD4 (2W1S) and CD8 (SIINFEKL) model antigens with or without 1 μg OMVs. Two weeks later, CD4 (**A**) and CD8 (**B**) T cell responses were assessed by FACS analysis using MHCcII and MHCcI tetramers, respectively. Total antigen-specific T cell numbers were calculated and compared using one-way ANOVA (*, *p* < 0.05; **, *p* < 0.01). Graphs present the mean + SEM for two independent experiments.

Activation of dendritic cells is the first necessary step towards development of cellular immune responses. Considering that OMVs effectively drive DC activation and maturation in vivo, we next tested whether OMVs could elicit cellular immune responses to co-delivered peptide antigens. First, we utilized the well-characterized 2W1S peptide, an I-A^b^ restricted epitope, to assess peptide specific CD4 T cell responses [26]. Mice were immunized with 2W1S peptide with or without OMVs, and antigen-specific CD4 T cells were measured by flow cytometry. As expected, immunization of mice with 2W1S peptide alone or a scrambled peptide control did not promote the development of 2W1S-specifc CD4 T cells (Figure 3A). However, immunization of mice with 2W1S peptide admixed with OMVs promoted a significant increase in 2W1S-specific CD4 T cells (Figure 3A). Next, we used the model MHCcI peptide SIINFEKL to assess CD8 T cell responses [27]. Once again, mice immunized peptide admixed with OMVs promoted a significant increase in peptide-specific CD8 T cells compared to mice immunized with peptide alone or scrambled peptide (Figure 3B). These data support the potential of OMV adjuvant to promote both helper and cytotoxic T cell responses to co-delivered antigens.

### 3.3. OMV Adjuvant Elicits B Cell and Antibody Responses to a Co-Delivered Protein Antigen

**Figure 4:**
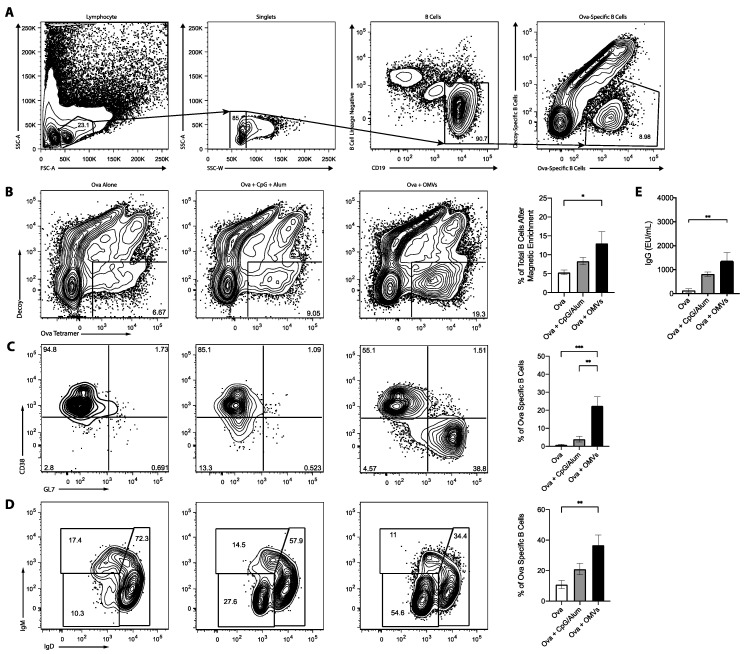
Mice (*n* = 3) were intramuscularly injected with either 10 μg of Ova alone, 10 μg of Ova plus 10 μg of CpG:alum (1:1), or 10 μg of Ova plus 10 μg of OMVs, and received a booster injections two weeks later. Mice were euthanized one week after the booster injection. (**A**) Representative gating strategy to show how lymphocytes, singlet, B cells, and Ova-specific B cells were identified. The spleens were harvested and stained with decoy and Ova tetramer (**B**), CD38 and GL7 to determine germinal center B cells (**C**), and IgM and IgD to ascertain the amount of isotype switching (**D**). (**E**) Ova-specific serum antibodies were detected by ELISAs. Statistics were performed using a one-way ANOVA with a Tukey’s multiple comparison test. *, *p* < 0.05; **, *p* < 0.01; ***, *p* < 0.001. Graphs present the mean + SEM for two independent experiments.

The ability of OMV adjuvant to elicit cellular immune responses to co-delivered antigens prompted us to examine antibody and B cell responses. For these experiments, we utilized the model protein antigen, ovalbumin, combined with the new OMV adjuvant or the well-characterized adjuvants alum and CpG DNA. As shown in Figure 4, mice that received Ova admixed with OMVs had a higher proportion of Ova-specific B cells in the spleen than mice that received Ova alone or Ova admixed with CpG:alum (1:1) (Figure 4A). Furthermore, the Ova-specific B cells from mice that were immunized with OMVs had an increase in B cells with a germinal center phenotype (Figure 4B) and had undergone isotype switching (Figure 4C) as compared to Ova alone or Ova plus CpG:alum. Consistent with the increased B cell activation, OMVs induced more anti-Ova IgG in comparison with Ova alone (Figure 4D). This demonstrates that OMVs, as an adjuvant, can produce a strong B cell response to co-delivered antigen.

### 3.4. Pre-Existing Antibody Does Not Inhibit OMV Adjuvanticity

**Figure 5:**
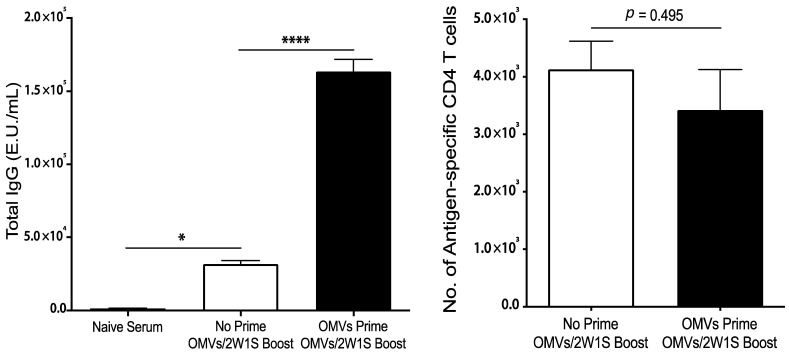
Mice (*n* = 3) were immunized subcutaneously with 1 μg of Bp82 OMVs on days 0 and 7, and then 1 μg of Bp82 OMVs + 200 ng 2W1S peptide on days 14 and 21. Control mice were given PBS on days 0 and 7, and then immunized the same on days 14 and 21 as the experimental group. On day 28, serum was harvested for ELISAs, and draining lymph nodes and spleens were harvested for T cell analysis. ELISAs were performed in duplicate by coating high-binding plates with 1 μg per well of Bp82 OMVs overnight at 4 °C. A mouse IgG standard was included on each plate. Plates were incubated with serial dilutions of immunized serum, and IgG responses were detected using a goat-anti-mouse total IgG-HRP secondary and developed using a TMB substrate. Plates were read at 450 nm and E.U./mL were calculated based off the Log EC50 of the standard curves on each plate. Groups were compared using one-way ANOVA (*, *p* < 0.05; ****, *p* < 0.001). The number of antigen-specific CD4 T cells were determined by flow cytometry using MHC-II tetramers (I-A^b^:2W1S-APC). Graphs present the mean + SEM for two independent experiments. T cell numbers were compared statistically using a Student *t* test.

The ability of OMVs to direct humoral and cellular immunity to co-delivered, unrelated antigens supports its potential utility as a stand-alone adjuvant. However, in order to function as an effective vaccine adjuvant, the immune response directed against OMV components must not interfere with OMV adjuvanticity. Thus, we next examined whether pre-existing antibodies specific for the OMVs impaired the cellular immune response to co-delivered antigen in a subsequent immunization. As shown in Figure 5, repeated immunization with OMVs did not impair antigen-specific CD4 T cell responses to co-delivered 2W1S despite the presence of high concentrations of OMV-specific serum antibodies.

## 4. Discussion

Development of new vaccine adjuvants that can enhance all arms of the immune response could have a major impact on overall vaccine efficacy. In this work, we showed that OMVs derived from an attenuated bacterium possessing a non-toxic lipid A are potent inducers of innate and adaptive immune responses. OMV nanoparticles were readily recognized and taken up by murine DCs, which subsequently led to the activation and maturation of the professional APCs. Notably, OMVs were able to drive DC responses at lower concentrations than heat-inactivated or live-attenuated bacteria in a side-by-side comparison both in vitro and in vivo. This is intriguing, as OMVs and bacterial preparations from the same source strain would be expected to contain similar pathogen-associated molecular patterns (PAMP). It is possible that OMVs presented specific PAMPs, such as lipopolysaccharide or surface lipoproteins, to the DCs in a more concentrated manner or that OMVs were better able to penetrate the cellular membrane, triggering both extracellular and cytosolic sensors, which enhanced DC activation. OMVs have been shown to signal through both TLR4 and caspase-11 [18,28], affirming the ability of OMV nanoparticles to access and stimulate both external and internal sensing pathways. Examination of the pathways responsible for OMV-mediated adjuvanticity was beyond the scope of the current study, and further work is needed to elucidate which pathways are essential for adjuvant function.

Another compelling finding in the present study is that OMVs, when simply admixed with an unrelated protein or peptide, elicited antibody, B cells, CD4 T cells, and CD8 T cells specific for the co-delivered antigen. This is highly significant because the totality of these immune responses is typically very difficult, if not impossible, to achieve without the use of a live or replicating vaccine. These responses were attained without directly linking or conjugating the antigens to the OMV. OMV delivery of the antigens to APCs could have occurred through non-specific or electrostatic interactions or through a depot effect. Other adjuvants, like alum, act in a similar manner to promote antigen uptake [29]. Alum, however, induces predominantly humoral immunity at the expense of cellular immune responses. Our data suggest that introduction of OMV adjuvant could potentially enhance cellular immune responses to a number of vaccine platforms, such as conjugate, subunit, peptide, VLP, and inactivated vaccines. Furthermore, the ability of OMVs to drive CD8 T cell responses would be especially advantageous for use in viral or cancer vaccines. OMV lipid-mediated membrane fusion with host cells, much like that described for escheriosomes [10], may account for delivery of antigens to the cytosol, leading to MHC class 1 presentation and CD8 T cell responses.

When compared at equal doses, OMVs were superior to an alum and CpG adjuvant combination in driving antibody and B cells in our experimental system. Use of combination adjuvants targeting different host pattern recognition receptors to achieve synergistic immune activation has been implemented for several licensed vaccines [30]. Alum combined with the TLR-9 agonist CpG is an especially potent combination adjuvant that has been widely utilized [31,32]. The ability of OMVs to outperform alum/CpG may be dependent on the route of immunization, since alum is primarily intended for intramuscular administration. OMVs exhibited adjuvanticity via multiple routes of parenteral administration, including intramuscular injection, which is the most common route used for traditional vaccines. OMVs also elicited adaptive immunity in vivo at very low doses (1 μg), which further attests to the potency of OMVs. This is an encouraging result because high OMV concentrations are more likely to cause cytotoxicity and unwanted reactogenicity.

OMVs are complex biological nanoparticles, containing numerous PAMPs and surface antigens recognized by immune cells [28]. As such, they constitute promising vaccine platforms as evidenced by the numerous studies reported to date in the scientific literature [8,33,34]. In order to assess the utility of OMVs as a vaccine adjuvant, it was important to determine if pre-existing OMV-specific humoral immune responses would interfere with OMV adjuvanticity. We showed that despite the presence of OMV-specific antibodies, OMVs were still able to drive cellular immune responses to a co-delivered peptide. While additional study is warranted, these data together with our observations from the DC experiments, suggest that OMVs rapidly impart adjuvanticity after in vivo administration. Incorporation of OMV adjuvant to new or existing vaccines could improve the magnitude and breadth of adaptive immune responses to enhance overall vaccine efficacy and represents an exciting new development in nanotechnology and vaccinology.

## Data Availability

Not applicable.
